# Mechanisms Underlying Mu Opioid Receptor Effects on Parallel Fiber-Purkinje Cell Synaptic Transmission in Mouse Cerebellar Cortex

**DOI:** 10.3389/fnsyn.2022.862704

**Published:** 2022-04-25

**Authors:** Yi Yang, Jin Bai, Jia-yue Sun, Ting Ye, Lu Zhang, Feng-ying Wu, Jun Nan, Yan Lan

**Affiliations:** ^1^Department of Physiology and Pathophysiology, College of Medicine, Yanbian University, Yanji, China; ^2^Interdisciplinary Program of Biological Functional Molecules, College of Integration Science, Yanbian University, Yanji, China; ^3^Department of Orthopedics, Affiliated Hospital of Yanbian University, Yanji, China

**Keywords:** mu-opioid receptor, parallel fiber, cerebellar Purkinje cell, synaptic transmission, miniature postsynaptic currents, protein kinase A (PKA)

## Abstract

μ-opioid receptors (MOR) are widely expressed in the brain, varying in density in different areas. Activation of MORs underlies analgesia, euphoria, but may lead to tolerance, dependence, and ultimately opioid addiction. The Purkinje cell (PC) is the only efferent neuron in the cerebellar cortex and receives glutamatergic synaptic inputs from the parallel fibers formed by the axons of granule cells. Studies have shown that MORs are expressed during the development of cerebellar cells. However, the distribution of MOR and their effects on PF-PC synaptic transmission remain unclear. To examine these questions, we used whole-cell patch clamp recordings and pharmacological methods to determine the effects and mechanisms of MOR activation on synaptic transmission at PF-PC synapses. The MOR-selective agonist DAMGO significantly reduced the amplitude and area under the curve (AUC) of PF-PC evoked (e) EPSCs, and increased the paired-pulse ratio (PPR).DAMGO-induced inhibitory effects on PF-PC eEPSCs and PPR were abolished by MOR specific blocker CTOP. Further, DAMGO significantly reduced the frequency of PF-PC mEPSCs, but had no obvious effect on their amplitude, suggesting a presynaptic site of action. The DAMGO-induced reduction in the frequency of PF-PC mEPSCs also was blocked by CTOP. A protein kinase A (PKA) inhibitor PKI added in the pipette solution did not affect the inhibitory effects on PF-PC mEPSCs induced by DAMGO. Both the PKA inhibitor K5720 and MEK inhibitor U0126 in artificial cerebrospinal fluid (ACSF) prevented the inhibitory effects of DAMGO on PF-PC mEPSCs. These findings reveal that MORs are expressed in presynaptic PF axon terminals, where DAMGO can activate presynaptic MORs to inhibit PF-PC synaptic transmission by regulating the release of glutamate. G-protein-dependent cAMP-PKA signaling pathway may be involved in this process.

## Introduction

There are three types of opioid receptors in the brain, the μ-opioid receptor (MOR), the δ-opioid receptor (DOR), and the κ-opioid receptor (KOR), mediating the actions of opioid drugs and endogenous opioid peptides (Zhang et al., 2019). Among these, the MOR shows the highest affinity with opiates and synthetic analogs to produce analgesia, euphoria, tolerance, dependence, and ultimately opioid addiction (Chartoff and Connery, 2014). The MOR is a seven-transmembrane-spanning, G-protein coupled receptor (GPCR) that signals *via* Gαi/o to suppress cyclic adenosine monophosphate (cAMP) formation by adenylyl cyclase (AC), and voltage-gated Ca^2+^ channels and stimulates G protein-activated inwardly rectifying K^+^ channels (GIRKs; Comer and Cahill, 2019). In recent years, studies have found that MOR activation also interacts with scaffolding proteins, such as β-arrestins, leading to ERK phosphorylation and other downstream events (Liu et al., 2015; Jean-Charles et al., 2017; Grim et al., 2020). Different agonists of MOR show different properties, for example, morphine binds to MOR and primarily activates signaling pathways through the activation of G-proteins, whereas fentanyl exhibits a signaling bias with greater β-arrestin relative to G-protein signaling (Jean-Charles et al., 2017; Comer and Cahill, 2019). Therefore, the design of clinical compounds based on different agonist signaling pathways has been proposed as a novel approach to enhance the therapeutic effects and reduce undesirable side effects in the protection of the cardiovascular system (Carr et al., 2016), the treatment of central nervous system related diseases (Park et al., 2016), and the regulation of inflammation (Galvani et al., 2015).

The cerebellum is an important region of the brain, playing a vital role in postural control, balance, and coordination of muscular activity. In recent years, numerous studies have demonstrated that it is also involved in cognitive and emotional functions such as language, learning, memory, fear, and reward (Attaai et al., 2020). As is well known, the cerebellar cortex of adult mammals contains three layers: a molecular layer, a Purkinje cell layer, and a granule cell layer, which are mainly composed of molecular layer interneurons (MLIs), Purkinje cells (PC), granule cells (GC) and Golgi cells (Harriman, 1974; Ruiz de Almodovar et al., 2010). Among these, the GCs are the most common type in the cerebellar cortex and their axons ascend into the molecular layer, form parallel fibers (Paredes-Ramos et al., 2011), running straight for several millimeters until they make synapses with PCs (Rössert et al., 2014). The PCs are the sole output from the cerebellar cortex to the deep cerebellar nucleus neurons, inhibiting them *via* γ-aminobutyric acid (GABA) released by their terminals (Paredes-Ramos et al., 2011). The PF-PC synapse in the cerebellum plays critical physiological roles and its involvement in motor learning and neurological diseases is well established (Selimi et al., 2009; Gallimore et al., 2016).

MORs are widely expressed in the brain, varying in density in different areas and playing different roles (Choi et al., 2008). Animal research in rodents indicates that MORs in GABAergic forebrain neurons modulate motivation for appetitive stimuli (Charbogne et al., 2017), MORs expressed in VTA are essential for alcohol reward-driven behaviors (Ben Hamida et al., 2019), and MORs may play a positive role in learning and memory by facilitating LTP in hippocampus CA3 neurons (Jamot et al., 2003). In humans, molecular imaging studies show that MOR availability is reduced in the striatum and other brain regions involved in hedonic processes and show increased cortical-subcortical correlations in schizophrenia (Ashok et al., 2019). Previous results from *in vivo* experiments showed that the MOR agonist fentanyl could inhibit the spontaneous and evoked firing activities of ML interneurons by activating MORs, and reduced the mouse cerebellum ML sensory information transmission induced by air-puff stimulation (Yang et al., 2020). However, the role of MORs in PF-PC synapses in the cerebellum remains poorly understood.

Therefore, we investigated the involvement and mechanism of MOR actions in PF-PC synaptic transmission in mouse cerebellar cortex by using whole-cell patch clamp recording technique and pharmacological methods in acute cerebellar slices.

## Material and Methods

### Subjects

Adult male Kunming mice weighing 28–33 g (aged 6–8 weeks) were used in all experiments and provided by the Experimental Animal Center of Yanbian University (SYXK (Ji) 2011-006). The experimental procedures were approved by the Animal Care and Use Committee of Yanbian University and were in accordance with the animal welfare guidelines of the U.S. National Institutes of Health.

### Drug Application

DAMGO, CTOP, KT5720, PKI were purchased from Tocris Bioscience (Bristol, United Kingdom), while picrotoxin, tetrodotoxin (TTX), and U0126 were purchased from Sigma-Aldrich (Shanghai, China). All chemicals were dissolved in artificial cerebrospinal fluid (ACSF) and they were applied to the cerebellar slices at 1 ml/min.

### Slice Preparation

The mice were anesthetized with isoflurane inhalation and quickly decapitated. The cerebellum was isolated and placed in ice-cold ACSF containing (in mM): 125 NaCl, 3 KCl, 2 CaCl_2_, 1 MgSO_4_, 25 NaHCO_3_, 1 NaH_2_PO_4_ and 10 D-glucose bubbled with 95% O_2_/5% CO_2_ (PH 7.4; 295–305 mOsm). The cerebellar sagittal slices (300 μm thickness) were prepared using a Vibratome (VT 1200s, Leica, Nussloch, Germany). The slices were quickly transferred to oxygenated ACSF for 60–90 min at room temperature (24–25°C) and the electrophysiological recordings began after incubation for ≥1 h.

### Electrophysiological Recordings

A Nikon microscope (Eclipse FN1, Nikon Corp., Tokyo, Japan) with a 60× water-immersion lens was used to identify the PCs in cerebellar slices. The recording electrodes and the stimulation electrodes were made using glass capillary tubes with an inner filament (od: 1.5 mm, Narishige, Tokyo, Japan). They were pulled at different temperatures using an automatic puller (PB-10, Narishige, Tokyo, Japan). Recording electrodes contained an internal patch solution composed of the following (in mM): K-gluconate 120, HEPES 10, EGTA 1, KCl 5, MgCl_2_ 3.5, NaCl_4_, Na_2_ATP 4, and Na_2_GTP 0.2 (PH 7.3 with KOH, osmolarity adjusted to 300 mOsm). Recording pipette resistances were 5 ± 1 MΩ in the bath and the series resistances were 15 ± 5 MΩ. To record the evoked excitatory postsynaptic currents (eEPSCs) of the PC the stimulating electrodes, which were filled with 20–30 μl ACSF and had an impedance of 0.1–0.5 MΩ, were placed on the molecular layer of the cerebellar slice. The PFs were stimulated with paired stimulus pulses at 0.5 Hz (0.2 ms duration, 10–100 μA intensity, 50 ms pulse interval). Membrane potential and current were monitored using an Axopatch 200 B amplifier (Molecular Devices, Foster City, CA, United States). The paired-pulse ratio (PPR) was calculated as the ratio of the current amplitude of evoked by the second pulse to that of the first pulse. The GABA_A_ receptors were blocked by adding picrotoxin (50 μM) in ACSF during the eEPSCs recordings. Miniature EPSCs (mEPSCs) were recorded at a holding potential of −70 mV in voltage-clamp mode and in the presence of TTX (1 μM) and picrotoxin (50 μM) to block voltage-dependent sodium channels and GABA_A_ receptors, respectively. The recordings, using Clampex 10.4 software (Molecular Devices, Foster City, CA, United States), were filtered at 5 kHz and stored on a personal computer using a Digidata 1550 series analog-to-digital interface. A step voltage command (5 mV, 10 ms) was applied to monitor series resistance. Cells with variable series resistance (>20%) were not included in the data analysis.

### Statistical Analysis

The Clampfit 10.4 software was used to analyze electrophysiological data. For analysis of mEPSCs, the Mini Analysis software (Version 6.0.3; Synaptosoft, Decatur, GA, USA) was used. The frequency and peak amplitude of mEPSCs were measured during a 120 s period and the threshold to measure mEPSCs was set at 3 pA in each condition. All calculated data are presented as the mean ± SEM. Statistical significance of data among different administration groups was determined by one-way ANOVA and Mann–Whitney-Wilcoxon test (SPSS Software; Chicago, IL, United States). *P-*value less than 0.05 was taken as a statistically significant difference.

## Results

### Effect of MOR Activation on Evoked Responses at PF-PC Synapses in Mouse Cerebellum

**Figure 1:**
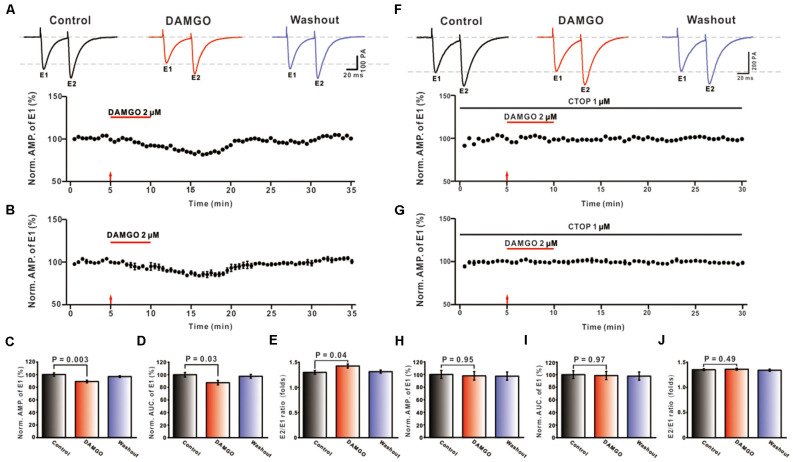
Effects of MORs activation on PF-PC eEPSCs. **(A)** Representative traces of PF-PC eEPSCs induced by paired-pulse stimulation in control, DAMGO (2 μM), and washout conditions (Top), and the time course of the DAMGO-induced changes in the amplitude of eEPSCs (Bottom). **(B)** Summarized results showing that DAMGO inhibited the evoked synaptic transmission of PF-PC in the cerebellar cortex (*n* = 8). **(C–E)** Bar graphs showing the effects of DAMGO (2 μM) on the normalized amplitude **(C)**, area under the curve (AUC, **D**) of E2/E1 eEPSCs paired-pulse ratio (PPR, **E**). **(F)** Representative traces of PF-PC eEPSCs induced by paired-pulse stimulation in control (1 μM CTOP), DAMGO (2 μM DAMGO + 1 μM CTOP), and washout conditions (Top) and the time course of the DAMGO-induced changes in amplitude of eEPSCs in the presence of CTOP (1 μM; Bottom). **(G)** Summarized results showing that CTOP blocked the inhibited synaptic transmission of PF-PC caused by DAMGO in the cerebellar cortex (*n* = 8). **(H–J)** Bar graphs showing the effects of DAMGO (2 μM) on the normalized eEPSC amplitude **(H)**, AUC **(I)**, and PPR **(J)** in the presence of CTOP (1 μM).

In order to examine the effect of MOR activation on synaptic transmission of PF-PC synapses in the mouse cerebellum, we first examined the effect of MOR agonist DAMGO on the eEPSCs. Recordings were performed in the presence of picrotoxin (50 μM), with the holding voltage set at −70 mV, and eEPSCs of PCs were induced by paired 0.5 Hz electrical stimulation of PF (0.2 ms duration, 10–100 μA intensity; 50 ms pulse interval). It was found that application of the MOR agonist DAMGO (2 μM) caused a reduction of PF-PC eEPSCs (Figures 1A,B). In addition, the reduction caused by DAMGO of the mean current amplitude was 11.12 ± 1.42% (DAMGO: 88.88 ± 2.01%, control: 100.00 ± 2.60%, *P* = 0.003 *n* = 8; Figure 1C) and of the AUC was 12.66 ± 1.96% (DAMGO: 87.34 ± 3.50%, control: 100.00 ± 3.54%, *P* = 0.03, *n* = 8; Figure 1D). To evaluate the site of DAMGO action at presynaptic or postsynaptic sites, the PPR value was further analyzed. The results showed that the mean value of PPR after the administration of DAMGO was 1.43 ± 0.04, which, compared to the control condition, was significantly higher (1.30 ± 0.04; *P* = 0.04, *n* = 8; Figure 1E). The above results indicated that the MOR agonist DAMGO inhibits PF-PC eEPSCs, accompanied by an increase in PPR value.

To further explore whether the DAMGO induced inhibition of evoked PF-PC synaptic response is directly mediated by MOR, in the presence of picrotoxin, the MOR specific antagonist CTOP (1 μM) and DAMGO were applied in conjunction. As shown in Figures 1F,G, the inhibitory effect of DAMGO on PF-PC eEPSCs was blocked by CTOP. Statistical analyses found that, in the presence of picrotoxin, the average amplitude of eEPSCs in the DAMGO condition does not significantly differ from the control condition (DAMGO: 97.83 ± 6.74%, control: 100.00 ± 6.33%, *P* = 0.97, *n* = 8; Figure 1H). Also, there was no significant difference between AUC (DAMGO: 98.58 ± 6.47%, control: 100.00 ± 4.72%, *P* = 0.97, *n* = 8; Figure 1I) and PPR value (DAMGO: 1.36 ± 0.02, control: 1.35 ± 0.02, *P* = 0.78, *n* = 8; Figure 1J) pre- and post-DAMGO perfusion. The above results demonstrated that the MOR agonist DAMGO may regulate glutamate (Bostanabad et al., 2021) release by activation of presynaptic MORs, thereby inhibiting the synaptic transmission at PF-PC synapses.

### Effect of MOR Activation on the mEPSCs of PF-PC in Mouse Cerebellum

**Figure 2:**
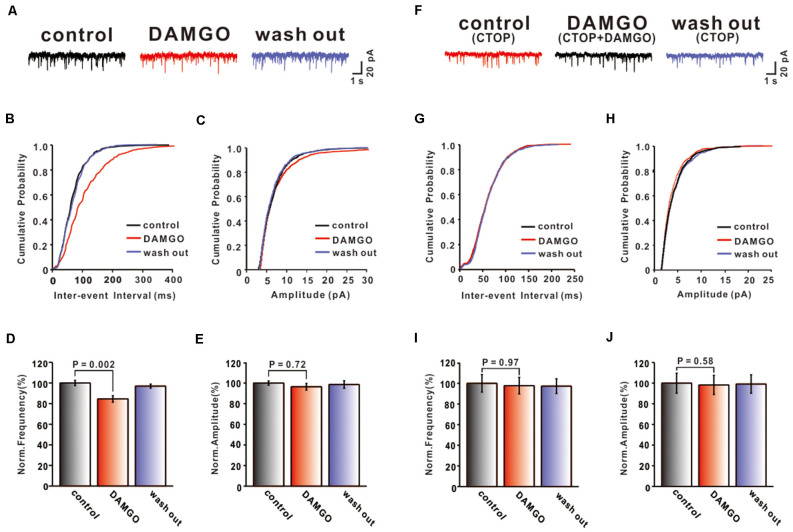
Effects of MOR activation on PF-PC mEPSCs. **(A)** Representative traces of PF-PC mEPSCs in control, DAMGO (2 μM), and washout conditions. **(B,C)** Cumulative probability plots of PF-PC mEPSCs frequency (interevent interval) **(B)** and amplitude **(C)** in control, DAMGO (2 μM), and washout conditions. **(D,E)** Bar graphs showing the effects of DAMGO (2 μM) on the PF-PC mEPSCs frequency **(D)** and amplitude **(E)**. **(F)** Represetative traces of PF-PC mEPSCs in control (1 μM CTOP), DAMGO (2 μM DAMGO + 1 μM CTOP), and washout conditions. **(G,H)** Cumulative probability plots of PC mEPSCs frequency (interevent interval) **(G)** and amplitude **(H)** in control (1 μM CTOP), DAMGO (2 μM DAMGO + 1 μM CTOP), and washout conditions. **(I,J)** Bar graphs showing the effects of DAMGO (2 μM) on the PF-PC mEPSCs frequency **(I)** and amplitude **(J)** in the presence of CTOP (1 μM).

To further confirm that DAMGO inhibits synaptic transmission by activating presynaptic MORs, the mEPSCs of PCs were also recorded. We set the holding voltage at −70 mV and perfused picrotoxin (50 μM) and TTX (1 μM) to block action potential generation and GABA_A_ receptors. As shown in Figure 2A, the application of DAMGO (2 μM) for about 5 min caused the cumulative probability of the inter-event interval curve to shift to the right, and the cumulative probability of the amplitude curve did not change (Figures 2B,C). This means that the frequency of mEPSC reduced significantly compared with pre-DAMGO application (control: 100.00 ± 2.47%, DAMGO: 84.52 ± 3.10%, *P* = 0.002, *n* = 6; Figure 2D) and the reduction of frequency was 15.48 ± 2.64%. However, perfusion of DAMGO did not change the amplitude of mEPSC (control: 100.00 ± 2.09%, DAMGO: 96.46 ± 3.20%, *P* = 0.72, *n* = 6; Figure 2E). These results indicate that DAMGO inhibited the synaptic transmission of PF-PC, under the blockade of inhibitory synaptic inputs and presynaptic action potentials.

We next examined whether the inhibitory effect of DAMGO is associated with MORs. CTOP (1 μM) and DAMGO (2 μM) were perfused in conjunction on the cerebellar slice. In the presence of picrotoxin (50 μM) and TTX (1 μM), we found that the inhibitory effect of DAMGO completely disappeared (Figure 2F). The frequency (control: 100.00 ± 8.49%, DAMGO: 97.79 ± 8.05%, *P* = 0.97, *n* = 6; Figures 2G,I) and the amplitude (control: 100.0 ± 9.66%, DAMGO: 98.27 ± 9.26%, *P* = 0.99, *n* = 6; Figures 2H,J) of mEPSCs did not differ compared with control conditions. These results demonstrated that DAMGO induced inhibition of mEPSC *via* activation of presynaptic MORs located on PF afferent fibers.

**Figure 3:**
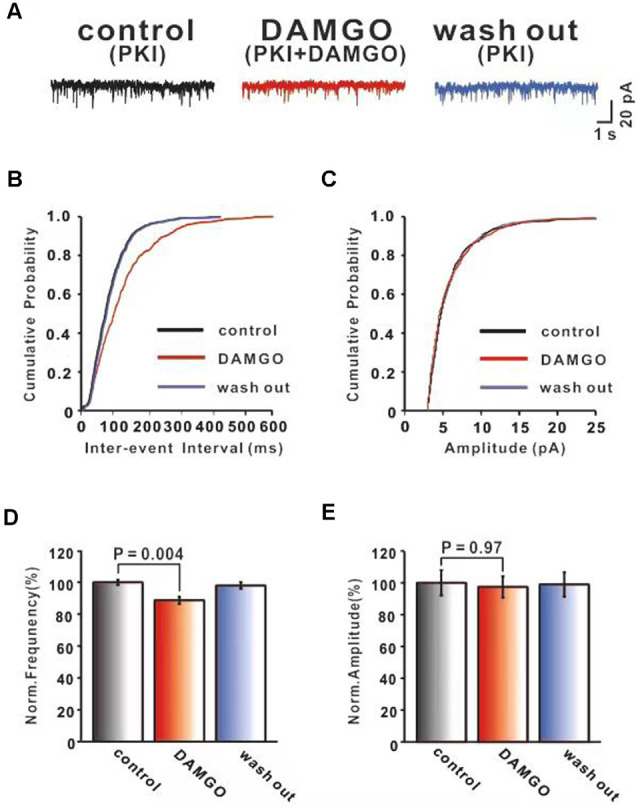
MOR activation inhibits PF-PC synaptic transmission after blocking postsynaptic MOR signaling pathway. **(A)** Representative traces of PF-PC mEPSCs in control (1 μM PKI), DAMGO (2 μM DAMGO + 1 μM PKI), and washout conditions (1 μM PKI). **(B,C)** Cumulative probability plots of PF-PC mEPSCs frequency (interevent interval) **(B)** and amplitude **(C)** in control (1 μM PKI), DAMGO (2 μM DAMGO +1 μM PKI), and washout conditions (1 μM PKI). **(D,E)** Bar graphs showing the effects of DAMGO(2 μM) on the PF-PC mEPSCs frequency **(D)** and amplitude **(E)** in the presence of PKI in internal solution.

To further test whether the inhibitory effects of DAMGO on the PF-PC synaptic transmission were mediated primarily by presynaptic MORs, we tested the effect of blocking MOR receptors in the postsynaptic neurons. It is well known that the MOR is a GPCR coupled with the Gαi*/*o subunits. Their activation reduces the content of cAMP and further stimulates the PKA signaling pathway inducing phosphorylation of downstream targets in the cytosol and nucleus. Therefore, the PKA inhibitor PKI (1 μM) was added to the internal solution to block this postsynaptic signaling pathway. In our experimental conditions, we usually began to record mEPSC 30 min after the internal pipette solution containing PKI started to diffuse into the cell, i.e., after rupture of the gigaohm seal. The effects of DAMGO on PF-PC mEPSCs were shown in Figure 3. It was found that in the presence of picrotoxin and TTX, the application of DAMGO still shifted the cumulative probability of the inter-event interval curve to the right (Figure 3B) whereas no change was observed in the amplitude of mEPSCs (Figure 3C). The statistical results showed that DAMGO decreased the frequency of mEPSCs (control: 100.0 ± 1.77%, DAMGO: 88.69 ± 2.38%, *P* = 0.004, *n* = 6; Figure 3D) and the amplitude of mEPSC (control: 100.0 ± 7.98%, DAMGO: 97.39 ± 6.74%, *P* = 0.97, *n* = 6; Figure 3E) did not differ compared with control conditions. Together, the data strongly suggest that the inhibitory effects of DAMGO on the mEPSCs recorded from PCs are mediated mainly by presynaptic MORs.

### Both PKA Inhibitor KT5720 and MEK Inhibitor U0126 Can Block DAMGO-Induced Inhibition of PF-PC Synaptic Transmission

**Figure 4:**
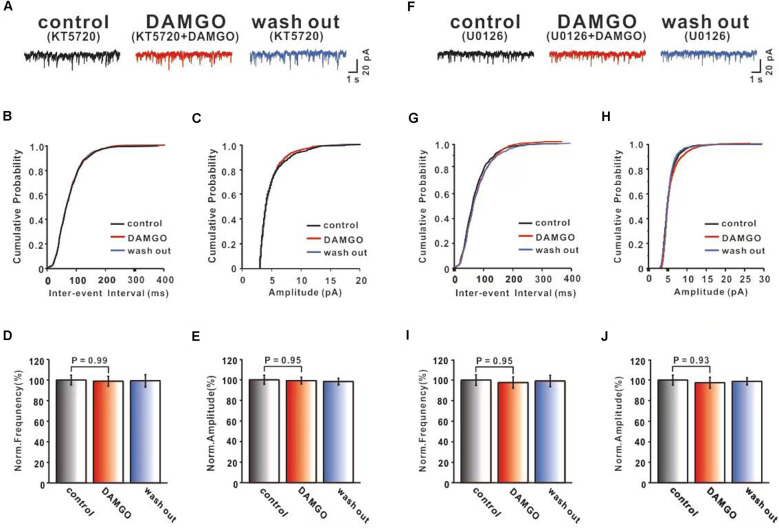
Both PKA inhibitor KT5720 and MEK inhibitor U0126 can block DAMGO induced inhibition of PF-PC synaptic transmission. **(A)** Representative traces of PF-PC mEPSCs in control (1 μM KT5720), DAMGO (2 μM DAMGO + 1 μM KT5720), and washout conditions (1 μM KT5720). **(B,C)** Cumulative probability plots of PC mEPSCs frequency (interevent interval) **(B)** and amplitude **(C)** in control (1 μM KT5720), DAMGO (2 μM DAMGO + 1 μM KT5720), and washout conditions (1 μM KT5720). **(D,E)** Bar graphs showing the effects of DAMGO (2 μM) on the PF-PC mEPSCs frequency **(D)** and amplitude **(E)** in the presence of KT5720 (1 μM) in ACSF. **(F)** Representative traces of PF-PC mEPSCs in control (10 μM U0126), DAMGO (2 μM DAMGO + 10 μM U0126), and washout conditions (10 μM U0126). **(G,H)** Cumulative probability plots of PF-PC mEPSCs frequency (interevent interval) **(G)** and amplitude **(H)** in control (10 μM U0126), DAMGO (2 μM DAMGO + 10 μM U0126), and washout conditions (10 μM U0126). **(I,J)** Bar graphs showing the effects of DAMGO (2 μM) on the PF-PC mEPSCs frequency **(I)** and amplitude **(J)** in the presence of U0126.

As a member of the GPCR family, MOR activation can inhibit cAMP generation *via* Gi/o proteins (Iftinca et al., 2020). It was reported that the major effect of cAMP as the second messenger signaling pathway is to activate cAMP-dependent protein kinase A (PKA), which phosphorylates the downstream target proteins influencing cellular metabolism, cycling, and growth. Previous studies showed that PKA regulated synaptic transmission through phosphorylation of presynaptic proteins, various ion channels, and neurotransmitter receptors (Jean-Charles et al., 2017; Comer and Cahill, 2019). In order to gain insight into the cAMP-PKA pathway and whether it is involved in the inhibition of PF-PC synaptic transmission by DAMGO, we applied the PKA inhibitor KT5720 (1 μM) on the cerebellar slice to investigate the effect of DAMGO on mEPSCs. We found that in the presence of KT5720 (1 μM), the DAMGO-induced decrease in the frequency of mEPSCs was completely prevented (Figure 4A). The normalized mean frequency of mEPSCs in DAMGO condition (KT5720 + DAMGO) did not differ from control condition (KT5720; control: 100.0 ± 4.83%; DAMGO: 98.91 ± 4.80%; *P* = 0.98 0.05; *n* = 6; Figures 4B,D). The normalized mean amplitude of mEPSCs in DAMGO condition (KT5720 + DAMGO) also had no significant change compared to control condition (control: 100.0 ± 4.40%, DAMGO: 99.26 ± 3.32%, *P* = 0.95, *n* = 6; Figures 4C,E). These results indicate that the DAMGO-induced decrease in the frequency of mEPSCs depends on the activation of the cAMP-PKA signaling pathway.

Alongside classical G protein pathways, activation of GPCR stimulates the β-arrestin signaling pathway, leading to MAPK/ERK signaling pathway phosphorylation and other downstream events (Liu et al., 2015; Eng et al., 2016). The ERK module of the MAPK cascade has also been implicated downstream of GPCRs, and evidence suggests that MOR-β-arrestin signaling promotes ERK phosphorylation in cell lines (Wheatley et al., 2012). Therefore, we selected ERK upstream kinase MEK non-competitive antagonist U0126 (10 μM) bath application to examine the effects of MORs. As shown in Figure 4, in the presence of U0126, DAMGO effects did not differ in terms of the frequency (control: 100.0 ± 5.16%, DAMGO: 97.62 ± 5.50%, *P* = 0.95% *n* = 6% Figures 4G,I) and amplitude (control: 100.0 ± 4.89%, DAMGO: 97.47 ± 5.32%, *P* = 0.93, *n* = 6; Figures 4H,J) compared with control conditions. These results demonstrate that the MAPK-ERK signaling pathway may participate in DAMGO induced inhibition of PF-PC synaptic transmission.

## Discussion

In this study, we examined the effects of DAMGO on PF-PC synaptic transmission in the mouse cerebellar cortex. Our studies demonstrate that activation of MORs reduces PF-PC eEPSCs amplitude and AUC, accompanied by an increase in PPR value. These results imply that DAMGO may exert actions on MORs at presynaptic sites. In order to confirm that DAMGO has a preferential presynaptic effect to inhibit transmission from PF to PC, two different experimental protocols were designed and showed that: (1) DAMGO significantly decreased the frequency, but not the amplitude, of mEPSCs recorded in the presence of TTX and picrotoxin; (2) the inhibitory effects of DAMGO on the mEPSCs remained unchanged when PKI was added into the internal solution to block G-protein mediated pathways in the postsynaptic neuron. These observations suggest that DAMGO produces presynaptic inhibition at the PF-PC synapse. To further explore the mechanisms of DAMGO induced inhibition in PF-PC synaptic transmission, we used PKA inhibitor KT5720 and MEK non-competitive antagonist U0126, respectively. The results showed that both KT5720 and U0126 blocked DAMGO-induced inhibition of mEPSCs, indicating that the cAMP-PKA signaling pathway and ERK signaling pathway may be involved in DAMGO-induced inhibition of PF-PC synaptic transmission.

MORs are widely distributed in the brain and expressed at different levels in different regions (Hwang et al., 2009). Results using *in situ* hybridization and immunohistochemistry showed that MOR mRNA was expressed most abundantly in the hypothalamus and midbrain, and to a lesser extent in the hippocampus and striatum of the rat (Bunzow et al., 1994; Zheng et al., 2005). Owing to the limitations of experimental methods, scientists once thought that MORs were absent in the rodent cerebellum. However, Mrkusich et al. (2004) found that MOR mRNA could be detected within PC, GC, and cells of the molecular layer in neonatal (P6) and adult rat cerebellum using exon-specific cRNA probes (Mrkusich et al., 2004). Recently, it was found that the cerebellum plays a key role in various non-motor functions such as arousal, emotion, and cognition, in addition to its well-established role in motor functions (Han et al., 2014). In the cerebellar cortex, PCs receive synaptic inputs from PF axons of GCs. PF-PC synaptic plasticity has been considered a primary cellular mechanism for motor learning and, because of its unique physiological properties, PF-PC synapses are also involved in neurological diseases (Selimi et al., 2009; Inoshita and Hirano, 2018). Many previous studies showed that opioids can increase the excitability of neuronal circuits by inhibiting the release of pre-synaptic vesicles in GABAergic neurons (Liao et al., 2007). Apart from GABAergic neurons, MORs also are present in glutamatergic neurons (Liao et al., 2005). Therefore, we explored the effects and mechanisms of MOR actions on PF-PC synaptic transmission in the cerebellar cortex of mice.

In our experiments, we first found that perfusion of MOR agonist DAMGO on cerebellar slices significantly decreased the amplitude, AUC, and increased PPR of eEPSCs in PF-PC synaptic responses. PPR is commonly used to indicate the direction and magnitude of changes in synaptic transmission and is widely used to determine the site of drug action at presynaptic or postsynaptic sites. PPR was calculated as the ratio of the initial eEPSC and the second eEPSC. Studies have found that when drugs act on the presynaptic compartment, the PPR value is often accompanied by significant or partial changes, that is, any alterations in PPR suggest a presynaptic site of action (Sun and Neugebauer, 2011). These results implied that activating MORs produced presynaptic inhibition at PF-PC synapses. We next recorded the mEPSCs of PCs in the presence of picrotoxin and TTX. Under conditions where action potentials are suppressed with TTX, the recorded mEPSCs are believed to reflect the quantal release from a single vesicle at one synapse (Hsu et al., 2003). As envisioned, DAMGO reduced the frequency of mEPSCs, but the amplitude did not change. CTOP could block DAMGO-induced inhibitory effects. These results raise the possibility that DAMGO may exert presynaptic effects by reducing the synaptic transmission of PF-PC. To exclude the possibility of postsynaptic MORs influencing synaptic transmission, we added the PKA inhibitor PKI into the recording electrode, which encompasses a PKA-specific inhibitory domain, to block the MORs of postsynaptic neurons. Consistent with the above results, DAMGO still increased the interevent interval of mEPSCs, and shifted the cumulative probability-interevent interval curve of mEPSCs to the right. All the above-mentioned results support our hypothesis that the MORs located on the presynaptic membrane of PF-PC synapses may be the main target of MOR agonist and antagonist regulation of function at PF-PC synapses. Activating MORs at PF-PC synaptic membranes can effectively inhibit synaptic transmission and decrease Glu release. Cramer et al. (2021) reported that activation of MORs with DAMGO suppressed mEPSCs in parabrachial nucleus (PB) neurons. Margolis and Fields (2016) also found that MOR selective agonist DAMGO inhibited a subset of LHb neurons by directly inhibiting Glu release.

The MOR belongs to the GPCR superfamily of seven transmembrane receptors and translates incoming extracellular stimuli into a variety of intracellular signaling cascades by regulating the Gα and Gβγ subunits of heterotrimeric G proteins (Hui et al., 2014). After activation by a ligand, conformational changes induce heterotrimeric Gαi/o proteins to the C terminus of the receptor. The GTP of the Gα subunit replaces the GDP and dissociates the G protein complex into Gα and Gβγ subunits (Stein, 2016). The Gαi/o proteins inhibit adenylyl cyclases and cAMP production, *via* the PKA signaling pathway to modulate the phosphorylation of downstream molecular targets. To test whether PKA activity was required for DAMGO-induced inhibition of PF-PC synaptic transmission, we added a selective PKA inhibitor, KT5720 to the ACSF. In the presence of KT5720, DAMGO did not change the frequency and amplitude of PF-PC mEPSCs. KT5720 is a widely used selective PKA inhibitor that binds to the catalytic subunits of PKA, acting as a competitive antagonist at the ATP binding sites on the catalytic domains and thereby inhibiting the phosphorylating activity of the kinase (Caruana and Dudek, 2020). The results suggested that the possible mechanism by which activating presynaptic MORs suppressed PF-PC synaptic transmission involved the cAMP-PKA signaling pathway. Xuan et al. (2018) also found similar results. Thus, inhibition of PKA abolished the effects of propofol on the PF-PC EPSCs, suggesting that propofol-induced depression of PF-PC EPSCs occurs through the PKA signaling pathway (Xuan et al., 2018). Seseña et al. (2014) also reported that MOR activation inhibits the I_Ca_ through the activation of a Gαi/o protein, which involves a decrease in AC-cAMP-PKA activity.

It is now well established that GPCRs signal not only through canonical heterotrimeric G protein-dependent pathways but also through G protein-independent β-arrestin signaling cascades (Liu et al., 2019). The agonist stimulation of GPCR leads to its phosphorylation followed by the binding of β-arrestin to GPCR, preventing further coupling of GPCR to G proteins and inhibiting GPCR signaling (Bostanabad et al., 2021). GPCR can induce different β-arrestin conformations (Lee et al., 2016). β-arrestin can combine with multiple protein kinases, resulting in functional signaling. Among these, the most representative signaling is MAP kinase (MAPK) cascades (Ras, Raf, MEK, ERK; Lee et al., 2016; Peterson and Luttrell, 2017). The Ras/Raf/MEK/ERK cascade has ERK as the last amplifier (Breitenbach et al., 2019). As we know, U0126 is a potent and selective non-competitive inhibitor of MEK, which has been widely used as a valuable pharmacological tool for studying the ERK signaling pathway (Wang et al., 2018). In our experiment, we used U0126 to block MEK, upstream of ERK, to inhibit the phosphorylation of ERK. We found that in the presence of U0126, DAMGO did not affect the frequency and amplitude of PF-PC mEPSCs. These results indicated that β-arrestin mediated MAPK-ERK signaling pathway may be involved in DAMGO induced inhibition of PF-PC synaptic transmission by inhibiting ERK phosphorylation. Zheng et al. (2010) reported that U0126 can block fentanyl-induced ERK phosphorylation in primary cultures of hippocampal neurons from β-arrestin2(−/−) mice.

A considerable amount of research indicates that different ligands can induce conformational reorganization of GPCRs, which allows the development of ligands to direct signaling toward different pathways, called “biased signaling”. It has been demonstrated that morphine, as the most commonly used analgesic in the clinic preferentially acts on MORs to activate G-protein signaling pathways to a greater extent than β-arrestin recruitment in the cell. However, fentanyl effects are biased toward β-arrestin recruitment (Jean-Charles et al., 2017; Comer and Cahill, 2019; de Waal et al., 2020). It is generally thought that DAMGO is a kind of unbiased MOR agonist. In our experiment, we found that both PKA inhibitor KT5720 and MEK inhibitor U0126 are effective at blocking the inhibitory effects of DAMGO on PF-PC synaptic transmission. These results indicated that both G-protein signaling and β-arrestin signaling may participate in the DAMGO induced inhibition of PF-PC synaptic transmission. However, we did not use β-arrestin knockout mice in this experiment. In addition, it was found that MAPK phosphorylation can be triggered *via* both G protein and β-arrestin pathways (Seyedabadi et al., 2019). Therefore, we did not clearly demonstrate whether the β-arrestin mediated ERK signaling pathway is involved in the mechanism of DAMGO induced inhibition of PF-PC synaptic transmission. Further studies in this regard will be undertaken in the future.

## Conclusions

In summary, we focused on the effects and mechanisms of MOR actions on PF-PC synaptic transmission in the mouse cerebellar cortex. Our findings revealed that MORs are expressed in presynaptic PF axon terminals, where DAMGO can activate presynaptic MORs to inhibit PF-PC synaptic transmission by regulating the release of presynaptic Glu. G-protein-dependent cAMP-PKA signaling pathway also may be involved in this process.

## Data Availability Statement

The raw data supporting the conclusions of this article will be made available by the authors, without undue reservation.

## Ethics Statement

The animal study was reviewed and approved by the Animal Care and Use Committee of Yanbian University.

## Author Contributions

YL conceived and designed the experiments and wrote the manuscript. YY and JB performed the experiments. TY, LZ, and JN analyzed the data. J-yS and F-yW contributed reagents and materials. All authors contributed to the article and approved the submitted version.

## Conflict of Interest

The authors declare that the research was conducted in the absence of any commercial or financial relationships that could be construed as a potential conflict of interest.

## Publisher’s Note

All claims expressed in this article are solely those of the authors and do not necessarily represent those of their affiliated organizations, or those of the publisher, the editors and the reviewers. Any product that may be evaluated in this article, or claim that may be made by its manufacturer, is not guaranteed or endorsed by the publisher.
